# Bio-Inspired Sutures: Localizing Damage by Isolating Strain Energy

**DOI:** 10.3390/biomimetics10020102

**Published:** 2025-02-11

**Authors:** Diana A. Chen, Melissa M. Gibbons

**Affiliations:** 1 Department of Integrated Engineering, University of San Diego, San Diego, CA 92110, USA; 2 Department of Mechanical Engineering, University of San Diego, San Diego, CA 92110, USA; mgibbons@sandiego.edu

**Keywords:** bio-inspired, suture, mechanical properties, finite element model, strain energy, local effects

## Abstract

This study draws upon bio-inspiration from anatomical sutures found in hard structures, such as turtle shells, to explore if impact energy can be dissipated through geometric parameterization rather than relying on energy-absorbing materials. While previous finite element analysis studies identified optimal dovetail suture geometries for maximizing the global stiffness and toughness of archway structures, this paper explores how different suture geometries might optimize localization effects through segmentation to isolate damage caused by the propagation of strain energy. We compare the global toughness of each suture geometry to its scaling factor, defined as the ratio of strain energy in the center segment(s) of the archway over the total strain energy absorbed during deformation, normalized by the expected strain energy consistent with uniform volumetric distribution. Our findings indicate that the scaling factor tended to correlate positively with global toughness, suggesting that suture geometries that performed well globally would also exhibit the localization effect. However, there is some nuance in selecting suture geometries that perform well for both metrics, as well as ensuring that geometries that perform well for one type of segmentation are still structurally sound in others, due to little control over where impact may occur, relative to the location of a suture, in real scenarios.

## 1. Introduction

The field of bio-inspired design leverages the solutions evolved by nature over billions of years, offering insight into engineering designs that incorporate characteristics and functionalities from biological structures to develop novel materials and structures that balance strength, flexibility, and durability [1,2,3,4,5]. One particularly compelling example is the role of anatomical sutures—natural interfaces found in many animals such as human skulls [6], turtle shells [7], and woodpecker beaks [8]—that connect stiff components (typically bone) with a softer material such as unmineralized collagen. These interfaces not only provide flexibility for growth and movement but also enhance resilience against sudden forces, providing impact resistance through energy dissipation mechanisms that prevent localized structural failures [9,10].

Engineering applications are often designed to optimize a specific mechanical property, like toughness, to ensure a structure’s ability to withstand unexpected forces without catastrophic failure. We are interested in the application of sutures to protective devices, such as helmets, where energy absorption and localization are critical to reducing injury risk [11,12]. The introduction of suture-like features has added a new dimension to such designs, enabling us to fine-tune energy dissipation through geometry rather than solely relying on advanced materials. Key to the effectiveness of bio-inspired sutures is the geometry of the interface, which governs the mechanical response of the overall structure.

Our long-term goal in this project is to create complex three-dimensional (3D) structures, such as helmets, using modular building blocks that have energy-absorbing properties. While the intended helmet application is hemispherical, we explore relatively simple curved archway structures under quasi-static out-of-plane loading as a first step toward optimizing the suture design for helmet applications. The archway structures are modeled as approximately the size of an average human head with the thickness of a typical bicycle helmet liner. Future work will investigate how the suture geometries can be expanded dimensionally to simulate high-speed impacts on more complex structures.

### 1.1. Literature Review

Several studies have been performed to fully characterize the complex hierarchical structure of the carapace (i.e., the outer shell) of the red-eared slider turtle and the corresponding mechanical properties of the individual components of the shell [7,13,14,15]. The 2D geometry of the sutures that are found between each pair of adjacent ribs has a zigzag shape [7,13]. Peng et al. found that the 3D microstructure of the suture is constructed by a pyramid-like shape; additionally, the hierarchical nature of the surface geometry led them to hypothesize that the increased contact area would increase the strength of the suture [15]. Krauss et al. tested samples under three-point bending and found that rib samples containing sutures behaved nonlinearly, with an initial low-stiffness response followed by a stiffening response under higher displacements that was activated after the two sides of the bone connected by the suture came into contact [7]; similar results were found by Achrai, Bar-On, and Wagner, with the additional finding that the samples failed when the protruding ‘teeth’ of the bony elements fractured [14]. Three-point bending tests performed by Peng et al. indicated that rib samples with sutures exhibited a lower bending strength than samples without sutures; however, the ribs with sutures sustained a much larger deformation, resulting in significantly more energy absorption before sample failure (i.e., increased toughness) [15].

The mechanical response of flat samples incorporating sharp sutures like the zigzag (triangular) sutures found in the turtle carapace, in addition to trapezoidal and rectangular sutures, has been studied under tensile loading [16,17,18]. Malik et al. defined critical geometric parameters for round dovetail sutures, including suture tab radius, contact angle, and tangent length, which together control the degree of interlocking and energy absorption of the resulting structure [19,20]. Xing et al. performed a similar study on elliptical interlocking sutures [21]. These works investigated the pullout response of single suture configurations in flat samples under tensile loading and established specific suture geometry parameter combinations that maximized the strength or toughness of the samples. Machine learning was used to optimize the design of a flat 2D sample incorporating a round jigsaw-like suture under tensile loading, demonstrating how artificial neural networks can be harnessed to explore a massive design space, allowing researchers to move away from a brute force approach [22]. Notably, they found that strength and stiffness were easier to obtain than toughness. Relatively few studies to date have examined the mechanical response of structures incorporating sutures under other loading conditions. Katz et al. developed and manufactured flat ceramic samples incorporating hierarchical suture shapes inspired by the white-tailed deer crania and diabolical ironclad beetle exoskeletons and performed tensile, four-point bending, and fracture tests [23]. They investigated how the sutures enhanced the toughness and flexibility of the otherwise brittle ceramic material. The flexural behavior of 3D-printed rectangular prism specimens with an interlocking ellipsoidal suture shape was investigated with three-point bending tests [24]. These findings underscore the potential for geometrically optimized suture structures in engineering applications where impact resistance and strain localization are critical, such as in helmet design. In all the studies summarized above, the samples themselves were flat; protective applications such as helmets, however, are curved. A recent study by Wu, Zhao, and Gao developed a computationally efficient interface-enhanced discrete element model to study the effects of three different interface geometries when incorporated into 3D protective structures mimicking a turtle shell, helmet, and a segmented space shield [25].

**Figure 1:**
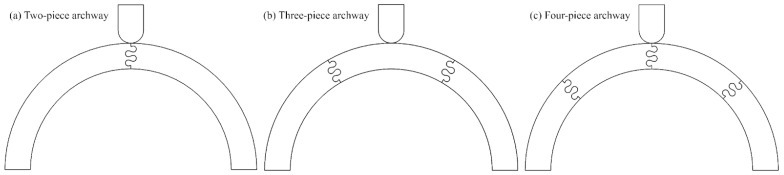
Archway structures were created with a varying number of component pieces. (**a**) Two-piece archway, (**b**) three-piece archway, and (**c**) four-piece archway. The suture parameters used in all three archways shown are a 20° contact angle, a 3 mm suture tab radius, and a 3 mm tangent length.

This foundational understanding inspired us to investigate how such parameters might enhance the toughness of segmented curved structures that can be incorporated into protective equipment. Our previous research used finite element models (FEMs) to examine the mechanical response of segmented archways with dovetail sutures under three-point bending. In a two-piece archway configuration, we found that the suture geometry with a 20° contact angle, a 1 mm tab radius, and a 2 mm tangent length achieved the highest overall toughness and stiffness [26]. We then examined the effects of archway segmentation [27]. The three archway configurations are shown in Figure 1. We found that the three-piece archway configuration, where the indenter was placed on a flat face rather than along a suture line, exhibited a higher resistance to sliding failure and was much less sensitive to changes in the specific suture geometry parameters. Four-piece archway configurations indented on the suture line proved to be the least stable, suggesting that segmentation must be optimized to balance stiffness, toughness, and structural stability. Our results underscore the complex relationship between suture geometry, segmentation angle, and indentation location in influencing a structure’s overall mechanical response.

### 1.2. Purpose of the Study

While we previously explored the total strain energy contained in the entire archway due to displacement [26,27], this study investigates how the placement of indentation affects the strain energy distribution within the archway structure, with particular emphasis on the strain energy localization effect for suture versus face indentation placements. We previously determined that increased segmentation of the archway is not beneficial for increasing the overall toughness of the archway (i.e., increasing global strain energy) [27], but hypothesize that increased segmentation may provide some benefits to the structure through the isolation of damage to select segments of the archway. This method of incorporating bio-inspired features to design for robustness has been extensively adopted in the structural engineering field [28]. We have taken the approach of rerouting loads across the structure using structural segmentation, a passive type of compartmentalization found in nature.

Consider three different archways: a solid archway, an archway composed of two pieces, and an archway composed of four pieces. In the solid archway, any impact energy is transferred throughout the piece and into the ground to which it is fixed. In the two-piece archway, the two archway pieces distribute the load evenly and again transfer the energy into the ground to which it is fixed. In the four-piece archway, however, the two additional suture lines act as barriers that prevent a portion of the load from being transferred into the ground to which it is fixed. In essence, the two indented archway pieces are ‘sacrificed’—the nearby sutures isolate the prospective damage to the system to a local area and salvage the rest of the structure. This effect could allow localized regions of the archway to absorb high strain energy, isolating the load from the adjacent segments and potentially minimizing damage to the entire structure. While we found that four-piece archways performed worse than both two- and three-piece archways for global stiffness and toughness (but followed similar trends exhibited by the two-piece archways) [27], this study explores if there is value to increased segmentation for localization effects rather than global properties. This paper explores the scaling factor of the localized strain energy based on the various suture geometries described previously.

## 2. Methods

The archway FEMs were created and solved in ANSYS Mechanical (V2022 R2, Canonsburg, PA, USA). Full details and in-depth rationale for all modeling decisions can be found in our previous studies [26,27], but a brief overview is provided here. The curved archway structure had an inner radius of 100 mm and a square cross-sectional area of 25.4 mm × 25.4 mm. Individual components revolved through a specified segmentation angle, denoted by Φ, were joined together with an interlocking dovetail suture. Archways composed of two pieces (Φ = 90°), three pieces (Φ = 60°), and four pieces (Φ = 45°) were created with this process.

The three geometric parameters that define the dovetail suture are (1) the suture tab radius, which controls the overall tab size, (2) the contact angle, which determines the degree of interlocking, and (3) the tangent length, which is a straight-line segment introduced between the protruding and recessing tabs, which increases the overall contact area between two connected pieces. We explored the entire range of physically admissible suture parameter values for the Φ = 90° archways [26]. Contact angles, θ, of 0–40°, suture tab radii, r, of 1–5 mm, and tangent lengths, L, of 0–20 mm were explored. We found that two-piece archways with a lack of a contact angle (θ = 0°), large suture tab radii (r > 3 mm), and large tangent lengths (L > 10 mm) produced suboptimal mechanical responses [26]. The response trends were similar for the Φ = 45° cases, so we excluded the θ = 0°, r > 3 mm, and L > 10 mm suture parameter values in the four-piece archways, and we sampled just a few tangent length values within each remaining contact angle/suture tab radius combination [27]. For the Φ = 60° cases, we explored the entire range of physically admissible suture parameter values because of the different loading condition. In cases where admissible geometries included a range of tangent lengths up to 20 mm, we simulated the tangent length in increments of 2 mm to adequately sample the full range [27]. Because we are interested in exploring how the presence of sutures contributes to localizing the global strain energy induced by the indenter’s displacement upon the midpoint of the archway, in the current study, we only analyze archways containing more than one suture. In total, this study comprised 188 simulations of Φ = 60° archways and 26 simulations of Φ = 45° archways.

**Figure 2:**
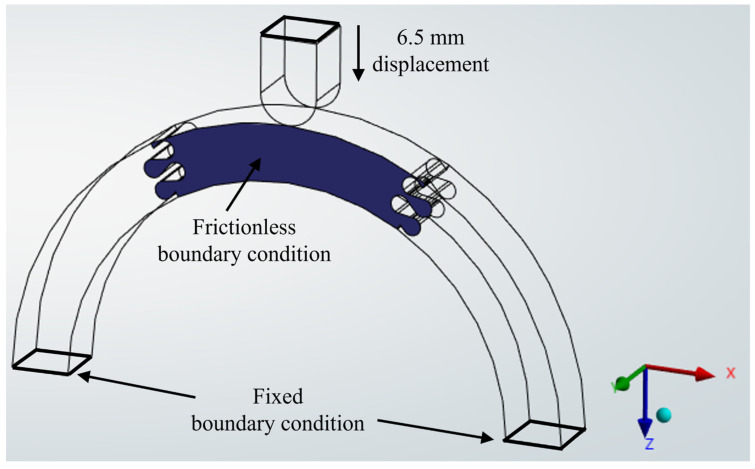
Representative Φ = 60° archway illustrating the boundary conditions and applied displacement.

The archway segments were assigned to be made of PLA plastic, which was chosen for future comparisons with 3D-printed experimental tests of the archways. The two faces at the feet of the archways were constrained by a fixed boundary condition, while a frictionless boundary condition was applied on one face of each center segment(s) in the x–z plane (see Figure 2) to prevent out-of-plane instability. All contact faces between segments were assigned to be frictionless. A quadratic mesh with a global element size of 3 mm was used on all parts, and contact sizing was applied on all contacting faces in the archway with an element size of 0.5 mm. Mesh convergence studies were performed to verify these mesh parameters were sufficient for the different archway configurations [26,27]. In the quasi-static displacement-controlled simulations, the archways were deformed through a 6.5 mm displacement of a steel indenter at the midpoint of the archway; this displacement induced substantial deformation in the archway pieces and visible suture tab pull out in many cases. In cases where the simulation failed to converge, we modified the mesh at the contact faces between the archway segments by reducing the contact sizing from 0.5 mm to 0.2 mm.

Our study focuses on the proportion of the overall strain energy stored in the archway segments not fixed to the ground. For Φ = 60° archways, which are composed of three archway segments with two sutures, the segments are equally divided into 33% by volume, while in Φ = 45° archways, which are composed of four archway segments with three sutures, the segments are equally divided into 25% by volume. We exported the total strain energy stored in the entire archway in its final deformed state and the strain energy stored in all archway segments not fixed to the ground, meaning the single center segment in Φ = 60° simulations and the combined strain energy stored in the two center segments in Φ = 45° simulations.

Consider the expected strain energy, e, to be consistent with the center segments’ overall volume: e = 0.33 for Φ = 60° archways and e = 0.50 for Φ = 45° archways. We defined f, the scaling factor, to be

(1) $$f=\left(s_{\mathrm{center}}/s_{\mathrm{total}}\right)/e,$$

where s_center_ is the strain energy in the center segment(s) and s_total_ is the total strain energy in the archway.

## 3. Results

### 3.1. Φ = 60° Archways

**Figure 3:**
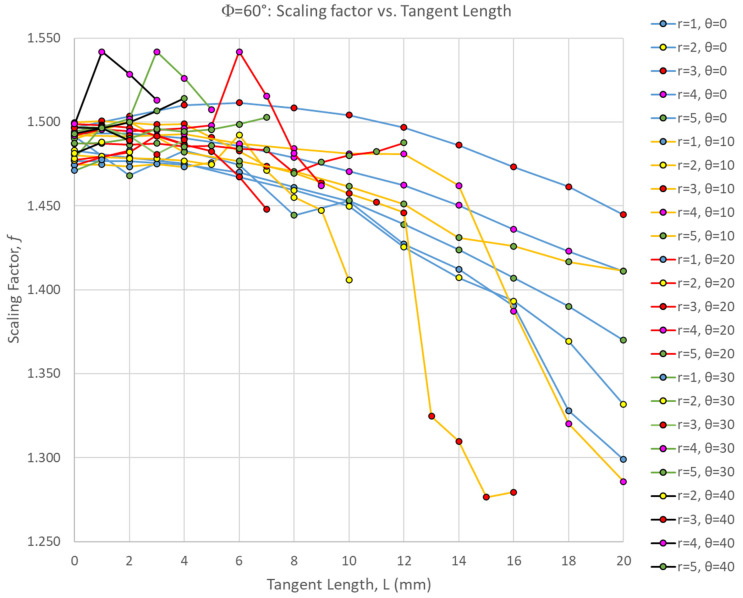
Scaling factor, f, as a function of the suture tangent length for all Φ = 60° cases. Each series represents a combination of the suture tab radius and contact angle used to create the dovetail suture that connects the three archway segments.

Figure 3 shows the scaling factor of all Φ = 60° simulations as a function of the suture tangent length, indicating a general downward trend as tangent length increases—similar to the global stiffness and toughness trends we observed previously [27]. However, there were non-monotonic trends in the scaling factor as all three suture geometry parameters were varied. The scaling factors are fairly tightly clustered together when there is no tangent length (L = 0 mm), and both increases and decreases in the scaling factor occur as the tangent length is increased. For the smaller contact angles of 0–10°, the scaling factor tends to decrease with increasing tangent length. There are exceptions to this trend, however; for example, when θ = 0° and r = 3 mm, there is a mild non-monotonic trend in which the scaling factor increases gradually before peaking at L = 6 mm, decreasing again as the tangent length continues to increase. For the larger contact angles, the scaling factor tends to generally increase with increasing tangent length. There are exceptions to this trend as well; distinct spikes in the scaling factor at intermediate tangent length values were found. For all contact angles, the scaling factor peaked when intermediate tangent lengths and suture tab radii were used: θ = 0° scaling factor peaked when r = 3 mm, L = 6 mm; θ = 10° scaling factor peaked when r = 3 mm, L = 1 mm; θ = 20° scaling factor peaked when r = 4 mm, L = 6 mm; θ = 30° scaling factor peaked when r = 4 mm, L = 3 mm; θ = 40° scaling factor peaked when r = 4 mm, L = 1 mm.

**Figure 4:**
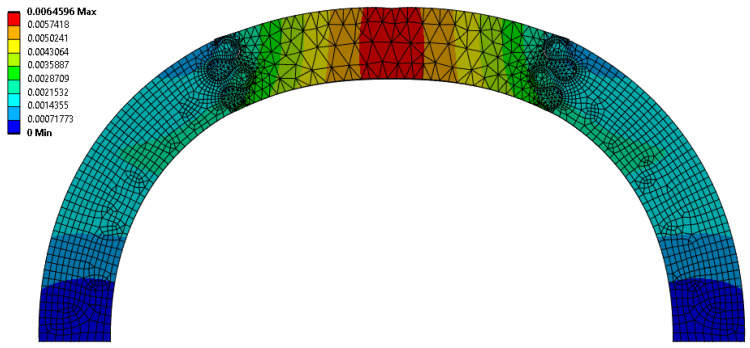
Total deformation (in mm, portrayed at the final indenter displacement of 6.5 mm) of θ = 30°, r = 4 mm, and L = 3 mm suture geometry, which resulted in the largest scaling factor (f = 1.542) of all Φ = 60° cases.

**Figure 5:**
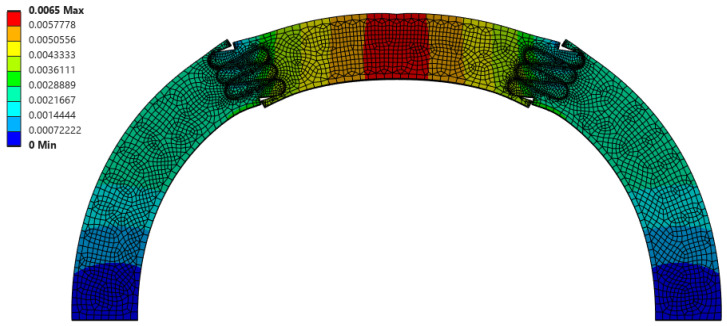
Total deformation (in mm, portrayed at the final indenter displacement of 6.5 mm) of θ = 10°, r = 3 mm, and L = 15 mm suture geometry, which resulted in the smallest scale factor (f = 1.277) of all Φ = 60° cases.

Interestingly, the scaling factor ranged from 1.277 < f < 1.542, with all cases being greater than 1.0 (the value expected if there was a uniform volumetric distribution of strain). This suggests that, even for the weakest suture geometries, the center segment of the archway did carry significantly more strain energy than the archway legs, indicating that the segmentation of the archway helped to isolate any potential damage from the rest of the structure. The suture geometry of θ = 30°, r = 4 mm, and L = 3 mm resulted in the largest scaling factor of 1.542 in the Φ = 60° archways. The final deformation of this suture geometry is shown in Figure 4. The suture geometry of θ = 10°, r = 3 mm, and L = 15 mm resulted in the smallest scaling factor of 1.277 in the Φ = 60° archways. The final deformation of this suture geometry is shown in Figure 5.

A comparison of Figure 4 and Figure 5 suggests that the increased interlocking (due to a high tangent length) in the sutures allows the center segment to flatten slightly, and in doing so, pushes the uppermost faces of the archway legs outward. In this case, more of the deformation imparted by the indenter is transferred to the archway’s legs, increasing the strain energy stored in those segments, thereby reducing the scaling factor. This explains why the scaling factor generally trends down as tangent length increases (Figure 3). In essence, the harder it is to separate the center segment from the rest of the archway, the lower we can expect the scaling factor to be.

**Figure 6:**
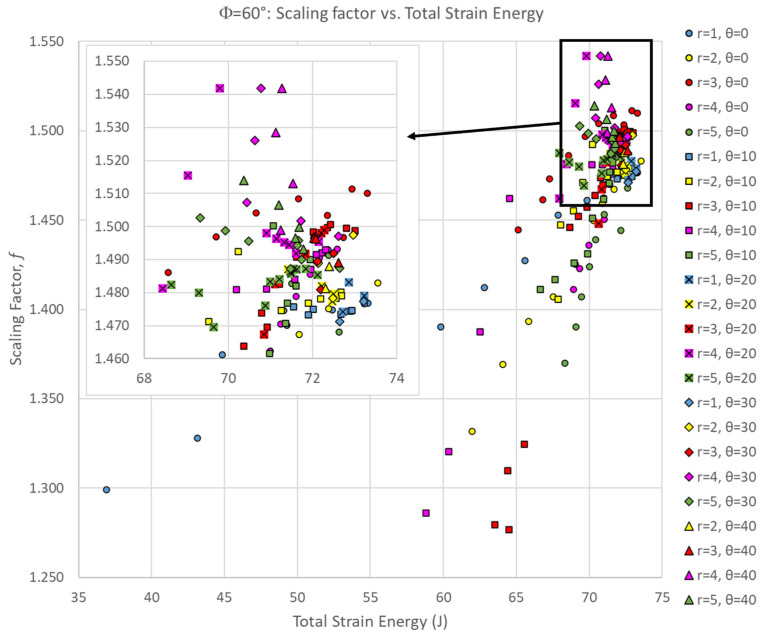
Scaling factor versus total strain energy for all Φ = 60° simulations. For clarity, the inset figure shows the portion of the dataset in the upper right-hand corner of the full dataset.

The scaling factor is only one part of the story; we want to identify suture geometries that are both globally tough (absorb the largest amounts of total strain energy) while also locally isolating as much of that strain energy as possible. Figure 6 shows a comparison of the scaling factor of the center segment to the total strain energy absorbed by the entire archway for all Φ = 60° simulations. In general, there is a positive correlation between the total strain energy and the strain energy localization effect. This suggests that suture geometries that increase the overall toughness of the archway structure are also beneficial for strain energy localization, which is an ideal outcome for the intended purpose.

### 3.2. Φ = 45° Archways

**Figure 7:**
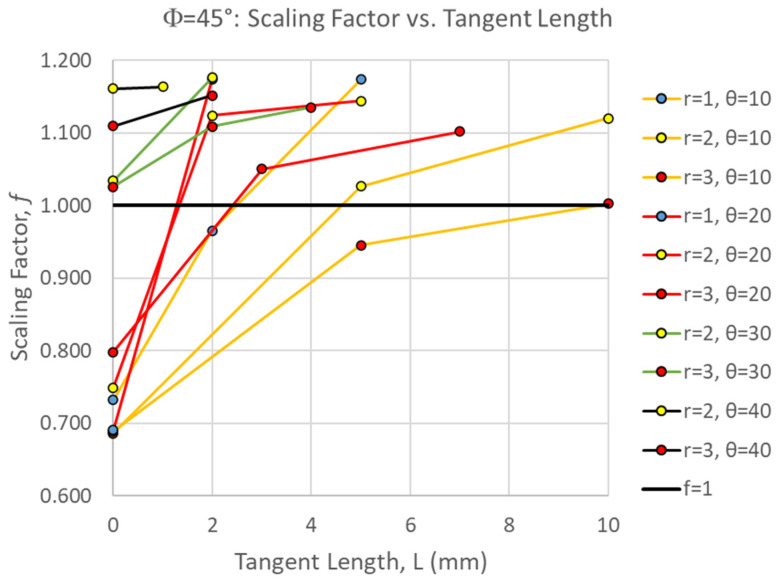
Scaling factor, f, as a function of the suture tangent length for all Φ = 45° cases. Each series represents a combination of the suture tab radius and contact angle used to create the dovetail suture that connects the three archway segments. Factors greater than 1.0 (line shown) indicate that there is strain localization in effect, where the center segments carry more strain energy than if the total strain energy were uniformly distributed by volume.

Figure 7 shows the scaling factor of all Φ = 45° simulations as a function of suture tangent length. The scaling factor ranged from 0.686 < f < 1.176. Factors less than 1.0 indicate that the two center pieces absorbed less strain energy than is expected proportional to their volume, contributing to worse damage to the archway legs. In general, the proportion of the strain energy localized in the two middle pieces increased with increasing tangent length, increasing contact angle, and decreasing suture tab radius (for a given contact angle and tangent length combination). To be more specific, the largest tangent length that can be introduced into a suture with a given tab radius and contact angle will result in the largest proportion of strain energy localization relative to a solid archway. Figure 7 illustrates that suture geometries with higher contact angles (θ = 30° and 40°), as well as longer tangent lengths at lower contact angles (L > 4 mm for θ = 10° and L > 2 mm for θ = 20°), resulted in scaling factors greater than 1.0. As a reminder, the physically admissible tangent lengths are limited as the suture tab radius decreases and the contact angle increases, providing a hard ceiling on the strain energy localization that can be achieved.

**Figure 8:**
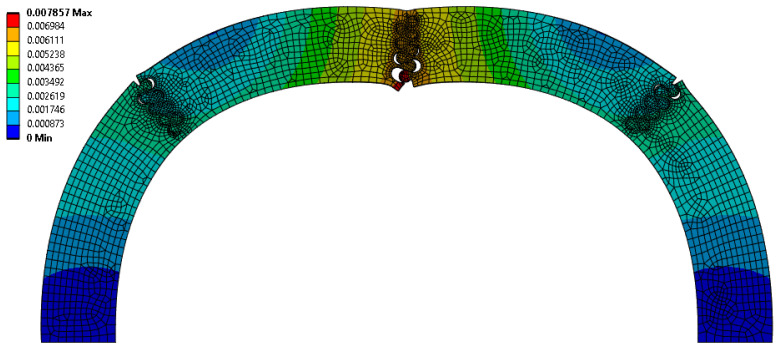
Final deformation of θ = 30°, r = 2 mm, and L = 2 mm suture geometry, which resulted in the largest scaling factor (f = 1.176) of all Φ = 45° cases.

**Figure 9:**
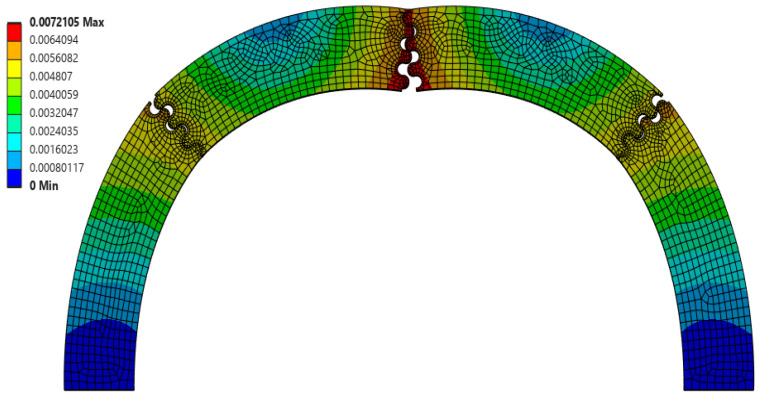
Final deformation of θ = 10°, r = 2 mm, and L=0 mm suture geometry, which resulted in the smallest scaling factor (f = 0.686) of all Φ = 45° cases.

The suture geometry of θ = 30°, r = 2 mm, and L = 2 mm resulted in the largest scale factor of 1.176 in the Φ = 45° archways. The final deformation of this suture geometry is shown in Figure 8. The suture geometry of θ = 10°, r = 2 mm, and L = 0 mm resulted in the smallest scale factor of 0.686 in the Φ = 45° archways. The final deformation of this suture geometry is shown in Figure 9.

A comparison of Figure 8 and Figure 9 suggests the opposite of what was observed in the Φ = 60° cases (as also indicated by the opposite trends in the scaling factor with suture tangent length shown in Figure 3 and Figure 7). Increased interlocking (due to a high contact angle) in the suture located at the indentation site results in the center pieces absorbing more strain energy before separating, which isolates the strain from the archway legs. Figure 9, with the smallest scaling factor for Φ = 45° cases, illustrates a more substantial separation of the center suture, which cascades into the next set of sutures due to the in-plane rotation of the center segments. As in the Φ = 60° archways, the larger deformation imparted to the sutures in the archway legs reduces the scaling factor. Therefore, the easier it is to separate the pieces connected at the center suture, the lower we can expect the scaling factor to be for Φ = 45° cases. 

**Figure 10:**
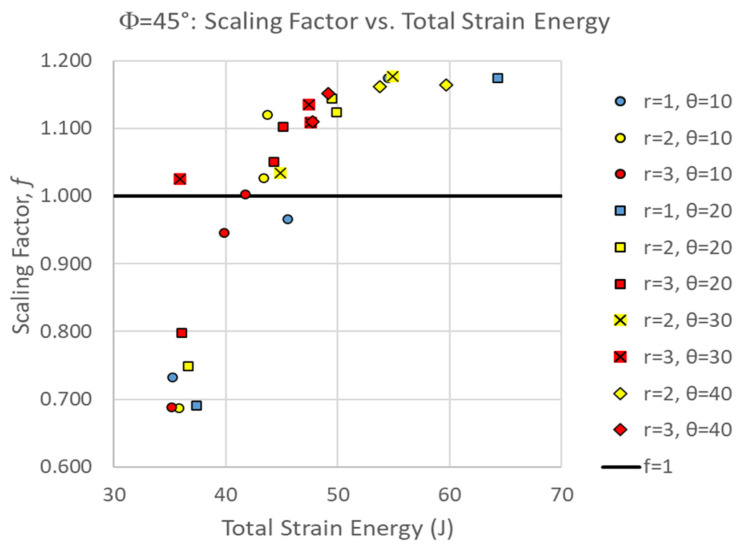
Scaling factor versus total strain energy for all Φ = 45° simulations illustrating a generally positive correlation.

Figure 10 shows an overall positive correlation between total strain energy absorbed (toughness of the structure as a whole) and the strain energy localization effect, which is the same trend that was observed in the Φ = 60° archways. In addition to indicating that both overall toughness and strain energy localization can be optimized with a select few suture geometries, the data also appear to begin to plateau, which suggests that there may be a maximum limit to the scaling factor in Φ = 45° configurations. Nonetheless, the positive correlation suggests that segmentation performed as intended for localizing damage to the structure while maintaining overall global toughness.

## 4. Discussion

### 4.1. Global Versus Local Toughness

In our previous investigations, we identified the ideal suture geometries for each of the various segmented archways for optimizing global stiffness and toughness [26,27]. However, we concluded that increased segmentation while holding the indenter placement constant (i.e., comparing Φ = 45° to Φ = 90° cases) contributed to worse overall performance, which led us to wonder about the merits of additional sutures in the archway [27]. The current study investigates the tradeoffs of localizing strain energy versus maximizing the global toughness of the different suture geometries.

In the Φ = 45° archways, most suture geometries with contact angles θ ≥ 20° produced some degree of strain energy localization (i.e., f > 1.0), and the larger contact angles tended to produce large scaling factors in the Φ = 60° archways as well, indicating that a large contact angle may be the dominant geometric parameter for optimal strain energy localization that accounts for impacts both on and away from a suture. In other words, when choosing an ideal suture geometry for helmet design, focusing on the contact angle (rather than the tab radius or tangent length) would guarantee that the scaling factor is always >1.0, in essence ensuring the preservation of the structure as a whole while containing any high strains due to impact to a local area.

The suture geometry θ = 20°, r = 1 mm, and L = 2 mm resulted in the highest global stiffness and toughness values for the Φ = 45° cases [27]. While this geometry did not produce the highest scaling factor (f = 1.176), it produced the second best, with a scaling factor f = 1.174 (99.8% of the maximum scaling factor), indicating this would be the best overall choice from both local and global perspectives. In comparison, if we prioritize the local metric, which resulted in the optimum geometry of θ = 30°, r = 2 mm, and L = 2 mm, its total strain energy s_total_ = 54.93 J is quite a bit lower than the maximum value of 64.269 J (which corresponds to 85.5% of the maximum total strain energy). Table 1 summarizes the optimal suture geometries based on their global and local strain energy metrics.

The suture geometry that optimizes both local and global toughness for Φ = 60° cases is a bit more nuanced. The suture geometry θ = 0°, r = 1 mm, and L = 0 mm resulted in the highest global stiffness and toughness values [27], but this only resulted in a scaling factor of f = 1.477, with f = 1.542 being the highest overall. While this scaling factor is certainly not on the lower end of the values produced (providing 95.8% of the maximum scaling factor), there are geometries that achieve higher local metrics while maintaining global metrics, indicating that this suture geometry would not be optimal for localizing impact energy. As we did above, if we instead prioritize the local metric, produced by a suture geometry of θ = 30°, r = 4 mm, and L = 3 mm, its total strain energy s_total_ = 70.776 J is 96.2% of the maximum total strain energy found in the Φ = 60° cases of 73.546 J.

Comparing the percentiles that each ‘best’ geometry falls under, Φ = 45° archways would be optimized by prioritizing global toughness, while Φ = 60° archways would be optimized by prioritizing local toughness. Ultimately, some other suture geometry may be better than any of the ones identified in Table 1. For instance, the suture geometry Φ = 60°, θ = 0°, r = 3 mm, L = 6 mm (an arbitrary geometry that rated highly on both axes in Figure 6) resulted in f = 1.511 (98.0% of the maximum scaling factor) with s_total_ = 72.93 J (99.5% of the maximum strain energy), which compromises both global and local metrics slightly but may be a ‘safer’ geometry overall. Before definitively selecting a suture geometry, whether one identified in Table 1 or another candidate like the one identified above, the results should be cross-checked across Φ (i.e., the response of the best Φ = 45° suture geometries should be checked in Φ = 60° archways, and vice versa). The suture geometries of the three high-performing Φ = 60° cases described above were not even simulated in the Φ = 45° archway because we had already discovered that small contact angles (i.e., θ = 0°) and large suture tab radii (i.e., r > 3 mm) produced suboptimal global responses [27]. However, the suture geometry identified as the best Φ = 45° case performs extremely well in a Φ = 60° archway. The suture geometry Φ = 60°, θ = 20°, r = 1 mm, and L = 2 mm, resulted in f = 1.484 (96.2% of the maximum scaling factor) with s_total_ = 72.868 J (99.4% of the maximum strain energy). Since there are a number of promising suture geometry candidates in the Φ = 60° dataset, this cross-checking process can be used to compare and even rule out two seemingly similar suture geometries.

Given these results, it becomes evident that, even with the broadly positive correlation between global and local toughness, there is a slight tradeoff between the local and global toughness metrics that affect which suture geometry is best suited for each type of archway. Selecting a suture geometry returns us to the original discussion of the applications of this study: is a globally stiff and tough helmet more valuable than one that can resist the transfer of deformation-causing loads?

### 4.2. Limitations

This study investigated whether, although increasing archway segmentation produced lower global stiffness and toughness metrics, there was value in increasing the segmentation of the archway for localization effects to isolate damage to the archway to individual segments. Our results indicate that a four-piece archway (Φ = 45°) never performed better than a three-piece archway (Φ = 60°) in terms of the strain energy scaling factor (i.e., the maximum f for Φ = 45° < minimum f for Φ = 60°). 

One limitation of this study is that when comparing Φ = 45° to Φ = 60°, the placement of the indenter is not held constant relative to the location of a suture, so we are unable to draw conclusions about performance due solely to increased segmentation. Our findings regarding four- versus two-piece archways suggest that symmetrically increased segmentation would result in similar trends but worse performance as segmentation increases [27]. However, because the two-piece archway (Φ = 90°) did not have enough segmentation to be considered in this localized strain study, there are insufficient data to conclude if degraded performance from three pieces (Φ = 60°) to four pieces (Φ = 45°) was due to increased segmentation or the placement of the indenter on a face versus a suture. Future work could consider expanding this study to examine five-piece archways (Φ = 36°), which would result in indentation on a face (comparable to the three-piece archway). This expanded study would provide information about increased segmentation (comparing three- and five-piece archways, both indented on faces), as well as information about the location of indentation (comparing three- and four-piece archways, and/or four- and five-piece archways). Following the trends identified in the comparison of four-piece to two-piece archways, we can assume that, globally, the five-piece archways would follow the trends of the three-piece archways, but at lower stiffness and toughness values. However, due to the placement of the indenter being an independent variable, we cannot know for certain whether five-piece archways would necessarily perform worse than four-piece archways. 

Another limitation of this study is the use of quasi-static impact loads rather than high-speed dynamic impact loads. Impact loading will produce stress waves that reflect off interfaces, and these dynamic effects must be thoroughly quantified to accurately predict the structural response. To complete the modeling phase of this work, high-speed impacts need to be simulated to confirm the optimal suture geometry and segmentation found in the quasi-static simulations perform well under conditions relevant to the final helmet application. The optimal suture geometry and segmentation combinations found from the simulation results will be used to produce experimental test pieces for high-speed drop tests to predict the injury reduction provided by a particular helmet design.

Based on our findings, we can conclude the following:

1. Increased segmentation (when holding the indenter placement constant) does not improve global performance in terms of stiffness and toughness [27].
2. Strain energy localization effects positively correlate with global toughness, indicating that the best suture geometries for global toughness are also adept for isolating damage to a local area, regardless of indenter placement. However, there is some nuance in selecting which suture geometries ensure better performance in both global and local metrics. Ideally, a geometry that is highly ranked in both global and local strain energy absorption should be selected. Of course, some segmentation is needed for localization effects to even be present; this presents a trade-off between global and local toughness, as global toughness is compromised when segmentation is increased.
3. Indentation on a face (Φ = 60°) rather than a suture (Φ = 45° or Φ = 90°) appears to improve both global and local performance in terms of stiffness and toughness. However, this external variable is unpredictable in a real-case scenario, i.e., a user would have no practical way to control where impact occurs on a helmet. This is why cross-checking results in different segmentation conditions is important; we found that optimizing based on the Φ = 45° performance is preferable, as the corresponding response (global and local) in the Φ = 60° archways tends to be quite good. This finding could be strengthened by further studies to standardize the process of suture geometry selection.
4. The “best” suture geometries in the Φ = 45° archway are only slightly compromised in the Φ = 60° archway, whereas the reverse is not true. Generally speaking, the Φ = 60° archway is less sensitive to suture geometry changes [27], both for global and local effects. Therefore, we should design around the impact on a suture, knowing that when we indent far away from a suture, the structure will still perform well.

## 5. Conclusions

Previous studies have investigated the role of bio-inspired sutures in improving the mechanical properties of different structures. The goal in determining the optimum suture parameters of a dovetailed suture, consisting of the tab radius, tangent length, and contact area, is to explore how a geometry, rather than the creation of new energy-absorbing material, can increase the overall toughness while maintaining the stiffness of a structure. In our previous research, we identified the optimal suture geometry for a simple, symmetric archway with a single suture that maximizes stiffness and toughness [26]. We subsequently studied the role of segmentation of the archway on the global mechanical response [27]. We explored the role of increasing segmentation on global toughness and found that increasing segmentation from two segments to four segments in fact worsened global toughness. While it is logical that global toughness would be worsened by introducing more “flaws”, there was also some merit to introducing more sutures to act as barriers to energy propagation. This led us to wonder whether increased segmentation would improve *local* toughness rather than global toughness. 

In this study, we hypothesized that segmented designs allow for interlocking components to act as “sacrificial” segments that protect the main structure by absorbing and dissipating impact energy, similar to natural sutures that disperse force while safeguarding the overall integrity of biological structures. We explored the localization of strain energy in the center segment(s) of symmetric archways to determine whether the addition of sutures contributed to the isolation of damage from the archway legs. We conducted the localization study on three-piece archways (Φ = 60°) and four-piece archways (Φ = 45°) and found that, similar to our global toughness findings, the four-piece archways did not perform as well as three-piece archways. However, future work is still needed, as the effects of segmentation and indenter placement cannot be assessed independently with these two datasets alone.

Insights from this study may support design strategies that incorporate bio-inspired sutures and structure segmentation to enhance both energy dissipation and damage isolation, with applications that extend to various fields of safety engineering. Future work will build upon these findings by evaluating the potential benefits and limitations of this strain energy localization effect under more complex configurations.

## Figures and Tables

**Table 1:** Best suture geometries and their global and local strain energy metrics when prioritizing one scale over the other.

	Best Global Suture Geometry	Best Local Suture Geometry
Φ = 45°	θ = 20°, r = 1 mm, and L = 2 mm	θ = 30°, r = 2 mm, and L = 2 mm
	f = 1.174 (99.8%)	f = 1.176 (100%)
	s_total_ = 64.269 J (100%)	s_total_ = 54.93 J (85.5%)
Φ = 60°	θ = 0°, r = 1 mm, and L = 0 mm	θ = 30°, r = 4 mm, and L = 3 mm
	f = 1.477 (95.8%)	f = 1.542 (100%)
	s_total_ = 73.316 J (100%)	s_total_ = 70.776 J (96.2%)

## Data Availability

The data that support the findings of this study are available from the corresponding author, D.A.C., upon reasonable request.
